# Medical Association of Texas

**Published:** 1854-05

**Authors:** 


### MEDICAL ASSOCIATION OF TEXAS.

We have before us a pamphlet containing the "Constitution and By-laws of the Medical Association of Texas," which was instituted in January 1853, and incorporated by the last Legislature of the State. The pamphlet also contains the proceedings of the last annual meeting, together with the anniversary address of the President, Dr. GEO. CUPPLES. Texas possesses the elements of a great State, and the proceedings of its Medical Society show that it is the determination of its physicians to advance the medical profession with the growth and development of the State. These proceedings would be creditable to the profession of any of the older members of the confederacy. The address of the President is forcible and eloquent.

